# PTEN in antigen presenting cells is a master regulator for Th17-mediated autoimmune pathology

**DOI:** 10.1186/ar3610

**Published:** 2012-02-09

**Authors:** Stephan Blüml, Gernot Schabbauer, Eva Hainzl, Birgit Niederreiter, Anastasia Hladik, Tobias Lohmeyer, Michael Bonelli, Elisabeth Zinser, Marije Koenders, Wim van den Berg, Giulio Superti-Furga, Josef S Smolen, Kurt Redlich

**Affiliations:** 1 Division of Rheumatology, Internal Medicine III, Medical University of Vienna, Austria; 2 Institute for Vascular Biology and Thrombosis Research, Center for Biomolecular Medicine and Pharmacology, Medical University Vienna, A-1090 Vienna, Austria; 3 Department of Dermatology, Hartmannstasse 14, University Hospital Erlangen, 91052 Erlangen, Germany; 4 Rheumatology Research and Advanced Therapeutics, Department of Rheumatology, Radboud University Nijmegen Medical Center, Nijmegen, The Netherlands; 5 CeMM - Center for Molecular Medicine of the Austrian Academy of Sciences, Vienna 1090, Austria

## 

Autoreactive T cells are a central element in many systemic autoimmune diseases. The generation of these pathogenic T cells is instructed by antigen presenting cells. However, signalling pathways in APC that drive autoimmunity are not completely understood. Here we show that that conditional deletion of PTEN in myeloid cells are almost completely protected from the development of two prototypic model autoimmune diseases, collagen induced arthritis (CIA) and experimental autoimmune encephalomyelitis (EAE). Myeloid specific deletion of PTEN lead to a significant reduction of cytokines pivotal for the induction of systemic autoimmunity such as IL-23 and IL-6 *in vitro *and *in vivo*. In addition, PTEN deficient dendritic cells showed reduced activation of p38 MAP-kinase and increased inhibitory phosphorylation of GSK3β in vitro. Dendritic cell and macrophage phenotypic maturation and migration to lymph nodes as well as collagen specific T and B cell activation was comparable in wt and myeloid specific PTEN^-/-^. However, analysing the impact of myeloid specific PTEN deficiency on T cell polarization, we found a significant reduction of a Th17 type of immune response characterized by reduced production of IL-17 and IL-22. Moreover, there was an increase in IL-4 production and higher numbers of regulatory T cells myeloid specific PTEN^-/-^. In contrast, myeloid specific PTEN deficiency did not affect serum transfer arthritis, which is independent of the adaptive immune system and solely depends on innate effector functions. These data demonstrate that the presence of PTEN in myeloid cells is required for the development of systemic autoimmunity. Deletion of PTEN in myeloid cells inhibits the development of CIA and EAE by preventing the generation of a pathogenic Th17 type of immune response.

